# AI, automation and the lightening of work

**DOI:** 10.1007/s00146-024-01959-3

**Published:** 2024-05-16

**Authors:** David A. Spencer

**Affiliations:** Economics Department, Leeds University Business School, University of Leeds, Leeds, UK

**Keywords:** AI, Automation, Work, Work quality, Work time, Future of work

## Abstract

Artificial intelligence (AI) technology poses possible threats to existing jobs. These threats extend not just to the number of jobs available but also to their quality. In the future, so some predict, workers could face fewer and potentially worse jobs, at least if society does not embrace reforms that manage the coming AI revolution. This paper uses the example of Daron Acemoglu and Simon Johnson’s recent book—*Power and Progress* (2023)—to illustrate some of the dilemmas and options for managing the future of work under AI. Acemoglu and Johnson, while warning of the potential negative effects of an AI-driven automation, argue that AI can be used for positive ends. In particular, they argue for its uses in creating more ‘good jobs’. This outcome will depend on democratising AI technology. This paper is critical of the approach taken by Acemoglu and Johnson—specifically, it misses the possibility for using AI to lighten work (i.e., to reduce its duration and improve its quality). This paper stresses the potential benefits of automation as a mechanism for lightening work. Its key arguments aim to advance critical debates focused on creating a future in which AI works for people not just for profits.

## Introduction

Artificial intelligence (AI) technology poses possible threats to existing jobs. These threats relate not just to the volume of jobs but also to their quality. A growing number of writers (e.g., Frey and Osborne 2017; Danaher 2019; Skidelsky 2024) warn of the above threats. They do so not in a way that implies the future is predetermined—rather, they suggest that society needs to mitigate the risks and realise the benefits of AI for work. If society is to avoid mass unemployment and enjoy the advantages of higher skilled and enriching work alongside more leisure time, then it will have to find ways to manage AI effectively.

In this paper, one example of recent research on AI and the future of work is examined critically. This example is the 2023 book, *Power and Progress*, by the economists Daron Acemoglu and Simon Johnson. Their book helps to elucidate some of the dilemmas and options for managing AI. Acemoglu and Johnson, as argued below, take a particular stance towards AI. They argue that it should be used to complement human labour. The risks they perceive with AI, from higher job losses to greater monitoring at work, can be reduced and a better future secured. The authors argue for a future where workers work with AI and where they enjoy the fruits of ‘good jobs’. This outcome, they argue, will depend on democratising AI technology.

This paper is critical of the approach taken by Acemoglu and Johnson—specifically, it misses the possibility for using AI to lighten work (i.e., to reduce its duration and improve its quality). This paper stresses the benefits of automation as a mechanism for lightening work. The danger with Acemoglu and Johnson’s approach is that it focuses on preserving work rather than on curtailing it. This is to suggest that people are mainly interested in maintaining their work lives and ignores their interests and needs in securing more time away from work. A more radical vision of the future would also include using AI to cut work hours and to extend leisure time. Ironically, this vision can be seen to be supported by the very arguments that Acemoglu and Johnson make in favour of expanding democracy in society. This paper contributes by offering a different account of how AI might be used to lighten work and to expand the opportunities for people to live well in all aspects of their lives. Broader critical implications are drawn at the end of the paper for post-work debates, the case for a universal basic income (UBI) and the goal of work time reduction. In all respects, this paper aims to advance thinking on the possibilities for using AI technology to create a better future of work, leisure and life.

In terms of the readership of this journal, this paper adds to past and current debates including on the effects of AI on work and society (see, for example, the recent symposium on ‘Embedding AI in society: ethics, policy, governance, and impacts’ edited by Pflanzer et al. (2023) and the critical review by Deranty and Corbin (2022) that cover some similar issues to this paper). It also takes inspiration from the approach of Mike Cooley (1987)—a founder of this journal—on ways that AI technology might be reshaped to create a better world, including one with a lower burden of work.

## AI and work: turning a threat into an opportunity

Acemoglu and Johnson (2023) offer a broad and detailed history of how technology has shaped economy and society over the last thousand year. Their book highlights the costs and benefits of technological progress. In particular, it illustrates the importance of power relations in influencing the direction of technology and the distribution of its rewards. While technology has contributed to ‘shared prosperity’, its ownership and control by particular economic elites has also fuelled inequality and brought direct harm to large sections of society. Its reform for progressive outcomes has therefore remained a key economic and political issue.

The authors pay particular attention to AI as a modern example of technology. They worry about biases in its development and use. In the work realm, in particular, there is too much emphasis on the use of AI for automation and monitoring. Tech-firms are developing AI that can automate work and disempower workers, while adopter firms are using AI to minimise the cost of labour and intensify work. With AI biased towards the creation of higher returns for a small number of investors and capital owners, the prospects for the sharing of the gains from technological change appear bleak. At least, they appear bleak under current socio-economic conditions. A key argument that Acemoglu and Johnson make is that change is possible and indeed necessary in creating a different and fairer economic future.

On the economic returns from AI, Acemoglu and Johnson strike a relatively pessimistic note. They are concerned that much AI technology is being used for what they term ‘so-so’ automation (Acemoglu and Johnson 2023, p. 19). That is, it is aiming to automate human labour without leading to any large (positive) productivity effect. Historically, technological progress has been associated with a so-called ‘productivity bandwagon’ effect (2023, pp. 14, 424). It has tended to boost productivity in a significant way, stimulating profit-maximising firms to expand output and to hire more labour. The increase in the demand for labour, in turn, has helped to raise real wages. The ‘productivity bandwagon’ effect has enabled both firms and workers to share in the gains from technological progress. AI technology in the present, however, is heavily biased towards automating human labour and has limited potential to create new tasks for workers to do. As a result, its wider use may simply add to unemployment and widen existing economic inequalities.

One particular example that Acemoglu and Johnson (2023, p. 19) focus upon is self-service checkouts. These automate the task of human cashier. In fact, they shift work rather than automate it—they transfer work from workers to customers. The incentive for employers to use self-service checkouts is that they save wage costs—for customers, they create work at the cost of their time. Acemoglu and Johnson argue that self-service checkouts do not create any great increase in productivity that can induce higher employment in other areas—in practice, they are no more productive than human cashiers. The introduction of self-service checkouts does not reduce the price of groceries, increase food production or change how shopping is undertaken. Rather, it merely substitutes for human labour and reduces direct employment. In the latter case, it makes it harder for workers to bargain for higher wages by creating an oversupply of labour. The above example, from Acemoglu and Johnson’s perspective, illustrates how AI can favour the interests of employers over those of workers and how its substitution for human labour may deliver unequal economic outcomes.

Beyond automating work, AI is also being used to monitor workers. Its use in this way intensifies work without any clear productivity dividend. It allows firms to reduce the wages they need to secure the same effort (i.e., it reduces ‘efficiency wages’). AI monitoring technology brings economic returns to managers and owners of firms, at the expense of higher work intensity and lower wages for workers. The most notorious example here are Amazon warehouses that have used AI to create workplaces that resemble ‘modern panopticons’. These workplaces, as Acemoglu and Johnson (2023, pp. 320–22) highlight, have been shown to be directly detrimental to the physical and mental health of workers. Their existence again shows the degree to which AI can be biased in its uses and how its extended application may not offer wider benefits to society.

The prevailing economic and institutional environment makes it extremely difficult for workers and the wider public to reshape and redirect AI. Tech-firms have evolved from small start-ups to large corporations, with dominant market positions. The wider corporate sector is governed by MBA-trained managers who are intent on maximising shareholder value, at the cost of lower wages and worse working conditions for workers (Acemoglu et al. 2022). Workers and their unions, for their part, are too weak to influence AI in ways that match with their interests. Ordinary citizens, finally, face disinformation through the use of AI on social media platforms, sowing the seeds of division and preventing forms of positive collective action.

Acemoglu and Johnson, like other modern authors (e.g., Zuboff 2019), reject a deterministic view of technological progress. Against those who argue that AI will likely bring about a work-less future (Susskind 2020), they stress how AI technology is malleable—society ultimately has choices over how it develops and uses AI.

There are different literatures that assert the malleability of technology. A longstanding radical literature deriving from Marx sees technology as shaped by capitalist social relations and as contributing to the exploitation of workers. Seminal contributions to this literature include Harry Braverman’s book (1974). They make the case for liberating technology from the profit imperative and for reusing technology under socialism (see also Marglin 1974; Cooley 1987; Caruso 2018). A less radical literature (Brynjolfsson and McAfee 2014; Schwab 2017)—one that Acemolgu and Johnson contribute to—emphasises the scope for different social actors to influence the nature and course of technology. It focuses less on the critique of capitalism than on the necessity and opportunity for reform under existing (capitalist) conditions.

A specific and urgent goal for Acemoglu and Johnson is to retain the human element of work. This suggests limits to AI that is directed at automation and monitoring. Instead, the focus should be on complementing human labour. It is implied that productivity will not be necessarily harmed by limiting the use of AI for automation and monitoring—in fact, it may even be improved.

First, as mentioned above, many AI applications may automate functions in a ‘so-so’ fashion (Acemoglu and Johnson 2023, p. 312)—limiting their use, therefore, may not lead to any great productivity loss. Second, AI may be more productive where it complements human labour. Examples from the health sector (e.g., radiology) show how the combination of AI and human workers can produce better results than fully automated alternatives (ibid.: 316) and demonstrate the merits of replacing automation with human-complementary technology.

Third, where AI is used for direct monitoring, it can impact negatively on workers’ motivation and morale. If employers focus on treating workers as mere instruments for getting work done and targets for cost reduction, they will lead workers to withdraw their goodwill and discretionary effort. The overall cost-savings for employers from AI monitoring technology may actually be small relative to the harm inflicted on workers (Acemoglu and Johnson 2023, pp. 322–323). Indeed, the use of AI for monitoring may crowd-out other routes to higher profitability that depend on enhancing the autonomy and skills of workers.

Acemoglu and Johnson (2023, p. 299) argue for AI that is ‘useful’. It should aim to ‘complement human capabilities and empower people’. They coin the term ‘machine usefulness’ (MU) (305) to signal a human-centred approach to AI. MU is consistent with increasing the productivity of workers in existing tasks and creating new tasks for workers to do—in this respect, it enables employment to remain at high levels. It also means using the information generated by AI to improve human decision-making. Workers may see their skills and knowledge enhanced rather than replaced by working with AI. Finally, MU fits with the expansion of economic platforms and markets, creating new employment opportunities and new sources of productivity growth (Acemoglu and Johnson 2023, pp. 327–330).

The concern, however, is that the benefits of MU are not being realised in the present, at least not to a significant degree. The reason is simple: ‘human-complementary machines are not attractive to organisations when they are intent on cost cutting’ (Acemoglu and Johnson 2023, p. 332). Reforms are, therefore, needed to push AI in a different and more positive direction.

The reform agenda proposed by Acemoglu and Johnson is multidimensional. First, it incorporates a change in ‘narrative’ about the future: ‘Debates on new technology ought to centre not just on the brilliance of new products and algorithms but also on whether they are working for the people or against the people’ (Acemoglu and Johnson 2023, p. 393). Investors should ask ‘whether new technologies will automate work or create new tasks, whether they will monitor or empower workers, and how they will affect political discourse and other social outcomes’ (ibid.). More generally, there must be a ‘new narrative about shared prosperity’ (394). This could be used directly to challenge the focus on shareholder value maximisation within modern organisations and to change business school teaching that is partly to blame for bad management. It could also be used to shift the attitudes of the young workers who are entering the tech sector.

The above narrative aims at using AI alongside human workers, with positive effects on labour productivity, task creation and human decision-making. It means broadening the purpose of AI and encouraging its use in ways that retain people in jobs that pay well and mean something to them.

Second, there is a need to build ‘countervailing power’ (Acemoglu and Johnson 2023, pp. 396–97). This means strengthening ‘worker organisation’. Historically, unions have offered a counterweight to the power of capital and a means to redistribute the proceeds of productivity growth. Union power has declined since the 1970s—a fact that is linked to the rise in economic inequality. Recently, however, there have been some signs of a revival in unionisation—unions have gained a presence, for example, in companies such as Amazon and Starbucks and used their influence to pushback against the exercise of employer power. Acemoglu and Johnson (2023, p. 398) encourage experiments in ‘new organisational forms’ as a way to redirect AI.

They also recommend ‘civil-society action’ (Acemoglu and Johnson 2023, pp. 398–99). While there remains a ‘collective action’ problem with incentives for individuals not to take action, groups of citizens can still mobilise and effect change, including in the direction of AI. New virtual public spaces for debate, indeed, can be created by digital technologies and could potentially add to political pressure for reform (400–402).

Third, there is a requirement for new government policies. These include ‘subsidies and support for more worker-friendly technologies, tax reform, worker-training programmes, data-ownership and data-protection schemes, breaking up of tech giants, and digital advertisement taxes’ (402). Added to this list are wealth taxes and a stronger social safety net to combat inequality (414–15).

What is noticeable here are the absences from the list. These include a UBI, which Acemoglu and Johnson dismiss directly. First, a UBI is more costly and less effective in tackling economic need than conventional taxes and transfers. Second, a UBI neglects the importance of work for well-being: ‘There is considerable evidence suggesting that people are more satisfied and are more engaged with their community when they are contributing value to society’ (416). A UBI cannot replace the psychological and social value of work. Third and more fundamentally, a UBI is ‘defeatist’ (417)—it accepts the inevitability of a future with less work when this future can be avoided by redirecting AI. Rather than redistribute income via a UBI, society should aim to ‘strengthen its existing safety nets’ while creating more ‘meaningful, well-paying jobs’ (417). As we will see below, perhaps surprisingly, one policy not mentioned at all is a strategy to reduce work time, even though this could be viewed as a desired outcome of technological progress as well as an efficient response to automation.

Two noteworthy aspects of Acemoglu and Johnson’s general approach can be singled out for attention. First, while rooted in a formal economic analysis, their approach is open to sociological and political economy considerations, not least around the balance of power between different interest groups and its influence on the direction of technology. Second, they promote what seems like a relatively bold reform agenda—one that recognises the need to challenge existing bases of power and to democratise AI technology.

In this second case, however, they face some tensions. While suggesting that the profit motive and shareholder value model are problems in terms of pushing AI technology in the wrong directions, they step back from recommending deeper reforms in the structure and governance of firms and the economy more generally. Rather, as the below discussion will show, they retreat to a rather conventional and conservative position that supports the continuation of paid work under capitalism.

## Why work matters (or making a case against automation)

One reason why Acemoglu and Johnson favour using AI to complement human labour is that it helps to keep people gainfully employed. This matters in two respects. First, it offers people a means to live. Protecting paid work is the surest way to prevent people from falling into economic destitution. The labour market remains the most effective mechanism for allocating income to people and is to be preferred over policies aimed at redistribution. Such policies include, as mentioned above, a UBI. Second, with paid work to do, people can secure the means to live well. Acemoglu and Johnson (2023, pp. 416–17) highlight the positive meaning of work and defend its promotion based on the direct value it brings to people’s lives. Human well-being is seen to depend on maintaining a high number of people in paid work.

The above view is distinctive, at least in economics. It goes against the standard view in economics that work is a ‘disutility’ at least on the margin. The idea that workers trade-off the marginal disutility of work against the marginal utility of consumption is deeply embedded in economics (Spencer 2009). This idea portrays work as an instrumental activity—a mere means to income—and disregards or downplays its role in shaping people’s lives. Indeed, it places the meaning of work effectively outside of economics. It offers support to creating less work rather than more work and backs technology that is focused on automation. Automating work and extending leisure time (a ‘good’) is seen as necessary and desirable in promoting well-being.

Acemoglu and Johnson adopt a different view, arguing that work influences how people achieve meaning in their lives (see also Susskind 2023). Economists are implored to see the direct value of work. This value includes the provision of wages, but it also extends to the status, recognition and self-esteem that workers get from performing work. These benefits will be missed if work is automated, and from the perspective of Acemoglu and Johnson, they cannot be offset by any form of redistribution policy.

Elsewhere, Acemoglu has written on the value of ‘good jobs’ (well-paid and secure). These are important not only in maintaining a positive motivation to work but also in fostering ‘civic and political participation’ (Acemoglu 2019, p. 2). He has argued that ‘good jobs are also necessary for society to enable a meaningful, fulfilling life for its citizens’ (2). Work defines people and provides a way for them to gain self-worth. If people could be compensated financially for not working (say by a UBI), this would be insufficient to make up for the loss of work itself. Automaton, on a mass scale, would destroy the meaning that many people get from working. As Acemoglu (2019, p. 2) puts it: ‘The prospect of a society in which few work (and enjoy the prestige and challenges of work) while many stay at home does not look enticing’.

Furthermore, he has argued that society must ‘generate meaningful employment opportunities—and thus a viable social purpose—for most people in society’ (Acemoglu 2021). This argument is motivated not just by a concern for the well-being of people but also by a desire to create a better functioning democratic system. Ensuring people have ‘good jobs’ is vital if democracy is to work effectively.

As we have seen above, Acemoglu and Johnson (2023, p. 417) support the creation of more ‘meaningful, well-paying jobs’. There are health reasons for backing their creation: more of them will prevent what Anne Case and Angus Deaton (2020) term as ‘deaths of despair’ (Acemoglu and Johnson 2023, p. 262). These deaths—due to excessive alcohol and drug use and higher suicide rates—have been linked to deindustrialisation in richer capitalist economies and provide a justification for creating more ‘good jobs’. Beyond preventing more deaths, however, such jobs will enable people to find meaning and purpose in their lives. They will help people to function as better workers, neighbours and citizens.

The point is that Acemoglu and Johnson are led to support maintaining people in employment because of a positive view of work’s meaning. Without this view, their case for supporting workers in employment would be weakened. Indeed, it would potentially disappear. If work is important just because of the income it provides, then no one would mourn its loss (assuming, that is, workers could be compensated with equivalent income). This is where advocates of a UBI can make headway and argue against calls for ‘more work’. Rather, the correct response to automation is to create other income sources, not to generate new sources of work (Danaher 2019). As has been pointed out already, however, Acemoglu and Johnson reject a UBI, partly because of its inability to substitute for the meaning that people get from work. Even with a UBI, people would suffer some loss of well-being and this loss can only be offset by providing work for them to perform.

The authors are not alone in arguing this point. Erik Brynjolfsson and Andrew McAfee (2014, pp. 234–35) make a similar argument. They see work as ‘beneficial’ for people and society. They allude to the psychological and social costs of joblessness as a penalty of automation—they refer to research that shows how US towns hit by manufacturing job losses have suffered both socially and economically and highlight evidence of the negative effects of unemployment on subjective well-being. They also stress the positive reasons (‘self-worth, community, engagement, healthy values, structure and dignity’) that motivate people to work and argue for ‘policies that encourage work, even as the second machine age progresses’ (Brynjolfsson and McAfee 2014, pp. 234, 236). For similar reasons as Acemoglu and Johnson, they reject a UBI because it does not meet people’s needs for work.

Like any generalisation, however, the argument that work is ‘meaningful’ or ‘beneficial’ remains contentious and open to direct criticism. Just as work can bring meaning and benefit to life, so it can degrade it and limit its scope to support well-being. What matters is how work is organised. There are no grounds for arguing that work must always be good and that its preservation must be given priority in all circumstances.

Indeed, on this last point, the hope has always been that technology would automate more necessary work and create more leisure time. Shorter work hours, in particular, have been achieved by automation—greater output has been secured with the same labour input due to technological progress. Productivity gains born of new technology have powered society towards shorter work hours (Huberman and Minns 2007). Automation has also lessened drudgery and increased the scope for meaning in work, though not without leaving some workers with hard and demeaning work to do. Cleaning jobs, for example, remain commonplace, despite state-of-the-art technology. Their existence reflects on the ease of employing lower paid (particularly female) workers and the limits to automation due to the non-routine nature of many low-paid tasks.

Against Acemoglu and Johnson, then, automation has often been viewed as an opportunity. It has been seen to bring forth the possibility of shorter work hours and less drudge work. Admittedly, there have been concerns that automation might take workers’ skills. The loss here may not just be via redundancy but also via lower quality work, as workers lose the ability to find meaning in work (Braverman 1974). From the Luddites onwards, there has been resistance by workers to machines that impair their skills; however, this resistance has been towards the uses of technology. The desire and demand for cuts in work hours and improvements in work quality has remained. Workers have not resisted automation per se but its use for goals such as deskilling (Cooley 1987). There has remained a latent and often unmet desire among workers for technology that lightens work (both quantitatively and qualitatively).

In the present, AI technology has represented some interests more than others. AI technology in the workplace, for example, has been used to meet the interests of consumers for more regular and cheaper services. Firms like Starbucks have used digital scheduling software to reduce labour costs directly (O’Neil 2016). Here, the interests of consumers have been put ahead of those of workers. In the latter case (using the example of Starbucks), workers have faced erosions in their control over work—customers have gained at their expense. The point is that there are different interests in play when AI technology is used and the meeting of some interests can be at the cost of others. When we consider that workers and consumers are mostly the same people, we can also see why the chief beneficiaries of AI technology are often tech firm bosses and other rich capital owners.

For the sake of this discussion, Acemoglu and Johnson recognise the interests of workers for meaningful work, but not their interests for work time reduction. The authors want to empower workers to find meaning in work, but fail to comment on how workers might be empowered to find meaning beyond work. Their focus on the positive sides to work leads them to ignore the benefits of using technology to reduce work time. They also miss the tensions between workers and employers over the duration of work time and how work time may stay long even while technology might be secured to shorten it. Again, the interests of tech firm owners and other wealthy capitalists may skew the goals of technology away from the interests of workers, only in this case the outcome may be longer work hours not just less meaningful work.

To address one of Acemoglu and Johnson’s concern, AI could prove ‘useful’ to workers in promoting shorter work hours. That is, it could help to secure for them more leisure time. Even ‘so-so’ automation may be beneficial if it helps to relief some workers of the drudgery of work while allowing them more time for themselves.

Acemoglu and Johnson express their desire for a ‘more balanced portfolio of innovations that complement human capabilities’ (Acemoglu and Johnson 2023, p. 395). This could include automation at least to some limited extent; this is why they reject direct taxes on automation (404). Their primary focus, however, remains on using AI to protect and promote paid work. Encouraging human-complementary forms of technology means retaining workers in full-time work. While some automation is not ruled out, the overriding goal is to keep people working and not to allow them the opportunity (and assumed lack of fulfilment) of a life with less work. The failure of Acemoglu and Johnson to see the interests that workers have in reducing work time and the potential to meet this interest via automation remains a major shortcoming.

To be sure, the authors allude, at times, to broader reforms. They refer, for example, to the merits of the German co-determination system in securing better outcomes for workers (Acemoglu and Johnson 2023, p. 404). They also refer tantalisingly to the possibility for ‘[s]elf-governance, both at the workplace and more generally’ (399).

Their main reference-point, however, is not a new society, but a model of shared capitalism along the lines of the one that existed in the post-war period. This model, which succeeded in achieving high growth rates with low levels of inequality, needs to be revived (or at least, its founding principles and goals need to be revived) if AI is to deliver for society. This mix of nostalgia for a bygone age and focus on maintaining paid work under a reformed capitalism blinds them to the possibility of a radically different future: one that could deliver shorter work hours potentially alongside more meaningful work. Their vision, ultimately, is too limited and not ambitious enough. As we shall see below, a different vision of the future (one supportive of automation) can be found in the work of past writers, most notably that of J.M. Keynes.

## Defending automation (or back to and beyond Keynes)

Acemoglu and Johnson intervene on a discussion that has been ongoing for a very long time. For centuries, economists have been debating when and if technology will sweep away jobs. Recurring bouts of ‘automation anxiety’ have occurred (Mokyr et al. 2015). Though on each occasion, jobs have tended to persist, defying predictions of the imminent demise of paid work. This fact reflects on the scope for jobs to advance with technology and on the barriers to automation. These barriers are partly explained by the ‘productivity bandwagon’ effect highlighted by Acemoglu and Johnson, though they can also be linked to the capacity of capitalism to sustain higher levels of consumption, fuelling and requiring more paid work. Employment has grown under capitalism, partly thanks to the rise in human wants and desires.

One notable contribution from the past is that of Keynes. His approach—set out in a famous 1930 essay—differs from that of Acemoglu and Johnson in some key respects. These differences help to clarify the basis of Acemoglu and Johnson’s arguments and the scope for their criticism and replacement.

Keynes wrote in 1930 that ‘technological unemployment’ would grow in the future. Society, he declared, was ‘suffering, not from the rheumatics of old age, but from the growing-pains of over-rapid changes, from the painfulness of readjustment between one economic period and another’ (Keynes 1963, p. 358). The capacity of the economy to maintain people in paid work would diminish over time, as technological progress accelerated. Keynes was full optimism about the future. While there would be significant disruption caused by the automation of paid work, coming years would bring about a higher standard of life. The reduction in working time—as the main prize of technological progress—would bring manifold benefits to society.

Keynes made the bold prediction that by 2030, the working week would fall to just fifteen hours. Beyond the economic depression of the 1930s, there was a brighter future. In this future, people would ditch the work ethic—instead, they would embrace a life of leisure and ease. Keynes wanted to see capital accumulation advance and backed state intervention to increase the incentive to invest. Full employment was needed not just to secure the livelihoods of workers but also to give them and their unions the bargaining power to press for shorter work hours. Keynes thought that full employment would provide the platform for a great reduction in work hours across the economy. In the transition to a shorter working week, society would be able to devote more of its time and energy to creative (non-work) activities, from painting to writing poetry. These activities would replace those of earning and spending money and would enable more people (workers and capitalists alike) to live ‘wisely and agreeably and well’ (Keynes 1963, p. 367). The future that Keynes envisaged translated, in form and content, to a post-capitalist environment: one in which the pursuit and possession of money would cease to exist. Instead, leisure would become the central pastime and preoccupation of human life.

Two contrasts between the essay of Keynes and the arguments of Acemoglu and Johnson can be highlighted. The first is that, unlike Acemoglu and Johnson, Keynes saw work as no more than a disutility. He saw little reason for people to work for its own sake—in fact, he took it for granted that people would want to give up working. The benefit of automation stemmed from the opportunity it afforded for people to live without work. A criticism here is that Keynes failed to see the intrinsic advantages of work and the possible welfare losses from the absence of work. He missed the point, rightly stressed by Acemoglu and Johnson, that work can have meaning and that its erosion through automation can harm well-being, at least where it replaces activities that people value.

Second, Keynes saw the automation of work as beneficial, because it extended time for creative forms of leisure. For Keynes, leisure was not idleness or the waste of time—rather, it entailed the creative uses of time. Set free from work, people would be able to develop and express their inner talents in all manner of activities. Unlike Acemoglu and Johnson, Keynes adopted a relaxed attitude towards work automation, believing that it would be vital for progress in society.

In this last respect, Keynes differed—in language at least—from other critics of work. Bertrand Russell (1935)—a contemporary of Keynes—gave praise to ‘idleness’ in an essay written in 1932. He lamented the fact that technology was being used to extend work rather than to reduce it and called for a rebalancing of society away from work. On closer inspection, however, Russell had in mind a similar utopia as Keynes. He wanted to see work diminish in order for people to be creative in activities of their own choosing. Russell equated utopia not with the expansion of laziness, but with the realisation of human creativity outside of work. He stressed the need for education to facilitate the wise use of leisure, but again, like Keynes, was confident that society could come to embrace and relish the freedom of a life without work. Russell wished for a different society, where the fruits of technology would be channelled, not into more work, but rather into more leisure.

The modern relevance of the ideas of Keynes as well as Russell is that they show the merits of pursuing automation as a way to reduce work time. In contrast to Acemoglu and Johnson, they urge us to think of ways to harness new technology that create more time for ourselves. This need not necessarily mean automating all work—again, Keynes and Russell were too dismissive of the meaning of work and overlooked the potential for meaningful work to exist. Rather, it is important that technology enhances not just the time for meaningful leisure but also the time for meaningful work. To achieve the twin goals of less and better work, however, we will need to contemplate radical futures: ones that push us beyond the ideas of Acemoglu and Johnson and necessitate reviving key critical content (often overlooked) in Keynes’ 1930 essay.

## Radically redirecting AI: for meaningful work and leisure

The reform agenda of Acemoglu and Johnson reduces, in essence, to supporting workers in high paid and secure jobs. They side with measures that will shift AI technology from automation and monitoring to the preservation and enhancement of paid work. They urge employers, workers, unions and the state to work in partnership to build a shared capitalism (similar to the model that existed in the years following the Second World War). That way, society can reap the benefits and combat the risks of AI.

In setting out their approach to reform, however, Acemoglu and Johnson are sceptical towards one particular reform, namely redistribution. ‘Building shared prosperity based predominantly on redistribution’, Acemoglu (2021) asserts, ‘is a fantasy’. This sentiment leads him and Johnson to reject a UBI, as we saw above. The basic argument is that the labour market can continue to play the role of redistributor. This is on the basis that ‘good jobs’ can be maintained into the future, provided automation is limited and offset by new job creation.

There are two problems here. First, redistribution was an important component of shared prosperity in the past. It is fanciful to think otherwise. In the post-war period, capitalist economies used progressive taxes to maintain low levels of inequality. Strong trade unions also helped to reduce inequality. The trend towards a more unequal capitalism since the 1970s has occurred on the back of the rolling back of progressive taxation and the decline of union power (Glyn 2006). Redistribution has and remains important in building more inclusive and equal societies.

Acemoglu and Johnson do not rule out redistribution entirely (the above quote includes the word ‘predominantly’ suggesting some redistribution might be permissible) but, aside from some general allusions to the German co-determination system of industrial relations, they fail to address concretely issues of democracy at work that could help to engineer a better deal for workers at work. They do not consider directly how workplaces might be reformed to secure for workers a stronger voice and stake in the process of AI development and usage and how such reform might be important in redistributing the rewards of AI.

Second, there is the fact that AI technology can be used to redistribute not just income but also time. Keynes’s vision, as we saw above, was to create a society where workers could use the bargaining power won by full employment to secure both higher wages and reduced work time. ‘Technological unemployment’ would be turned from a threat into an opportunity through the economisation of the human participation in work. The reduction of the average working week would be the result of capitalism. In the transition to a shorter working week, however, life would be transformed. Instead of spending time in search of money for consumption, people would be freed to live as they wanted to. Redistribution of time from work to leisure in this case meant an improvement in the quality of life. It would also hasten the demise of capitalist ways of living and facilitate the move beyond capitalism.

This latter aspect plays no part in Acemoglu and Johnson’s book. When referring to Keynes, they invoke his ‘fears’ about technological unemployment but not his hopes for a better (post-capitalist) future (Acemoglu and Johnson 2023, pp. 11–13). They are generally content to stick with capitalism rather than contemplate a future beyond it. They overlook how an AI-driven automation might be a means to transform society beyond the work-centred way of life that exists now.

Clarity is needed on two points. First, the goals of automation. For past thinkers like Keynes and Russell, the replacement of work with leisure was the main goal. It can be argued here, following Keynes and Russell, that a goal of automation should be to deliver less work. This is on the basis that more time away from work is needed for people to meet and realise their creative needs (in this case, by pursuing self-determined activities outside of work).

However, there should also be a role for automation in improving work’s quality. This brings in some of the arguments made by Acemoglu and Johnson but it also goes beyond them. The point is that work can vary in its meaning. Acemoglu and Johnson place stress on the personal and social utility of work—they treat work as a means for people to gain self-worth and to feel a part of society. In a world where work is lessened through automation, however, people may begin to gain self-worth and feel a part of society through activities outside of work. Indeed, this may be viewed as a healthy development and as a mark of progress in society. Relative to the situation where work monopolises large parts of people’s lives, it may be seen as a good thing that more people can find a life and a means for enjoyment beyond work. In short, norms may adjust to valuing a life that is not just focused on continuous work. The criticism of Acemoglu and Johnson is then that they miss how automation might be used to promote alternative ways of living that allow for well-being outside of work.

That said, work still has its place in meeting people’s needs. Work’s normative value may be challenged in society as automation accelerates, but its roles in meeting human needs for sociality and creativity may persist. Indeed, these roles may lead workers to miss their jobs even if they are granted extended leisure time and/or income compensation. From an opposite direction, workers may be doubly benefitted by automation if it reduces work hours and frees them from the drudgery that their jobs entail.

The above arguments allow us to rethink automation as a mechanism for expanding human freedom in two realms. First, in the realm of leisure, where people can choose to act as they wish. Second, in the realm of work, where people can be granted not just more freedom from toil but also more freedom to work well. The point of technology, including AI, is not then to keep people working in regular jobs, but to transform the work and leisure lives of people, so that they can feel at home in work as well as outside of it. This latter vision of automation retains the radical intention of Keynes to create a leisure society, but also extends it by allowing room for work in the conception of a good or better life. Acemoglu and Johnson, for their part, are right to focus on work’s value, but wrong to assert that it must be maintained seemingly at the expense of automation. What is required instead is automation that promotes both meaningful work and leisure.

The second point relates to the scope for reform. The demand for lighter work—in a quantitative and qualitative sense—requires significant changes in the structure of the economy (Spencer 2022). In particular, as is implied by Acemoglu and Johnson, it requires challenging prevailing motives and practices within organisations that place profits ahead of people. Yet, taking this point seriously means contemplating changes that Acemoglu and Johnson are reluctant or unwilling to embrace. In short, a profit-driven AI may remain fundamentally incompatible with a human-centred AI (Christiaens 2024). Reforms may moderate and check the costs of a profit-driven approach but not fully resolve them. Rather, the pursuit of profits may mean that AI technology is inherently biased against workers and society more generally.

As AI technology is linked to specific expertise and the centralisation of data (including the collection of masses of personal information), so the scope for it to be governed in undemocratic and partisan ways is increased. The wider point is that AI technology raises deep issues over governance and accountability. To the extent that it is operated for profit, the scope for it to do good in society is limited, at least without significant change in the way that the economy is structured and organised.

Acemoglu and Johnson recognise the dangers of AI but show political naivety in thinking that these dangers can be overcome through a set of well-targeted reforms. They miss the need for shifts in ownership that would lead to a more radical change in the way that work and technology are governed. The case for workers taking ownership of productive assets and directing AI is missed by Acemoglu and Johnson, even though it may be regarded as a crucial step in humanising work and technology.

In essence, for AI to work for society, the economy needs to be rethought (Cooley 1987). The objective of the economy needs to switch from making more money to creating the conditions for human flourishing in all spheres of life. AI technology raises some particular issues—it cannot be thought simply as a tool for serving human needs, since it has the capacity to ape human skills. In this sense, the goal may be for humans to collaborate with AI rather than to master it. This goal, however, could still go along with human workers producing with skill and meaning. The point is that the economy must become an arena where workers’ interests for meaningful work together with ample leisure time are respected and upheld in the development and use of AI technology.

The question of whether the above means thinking beyond capitalism can be raised. It may be asked more directly whether AI technology can ever be human-centred until and unless capitalism is transcended. Keynes, while supportive of capitalism in the short-run, envisioned a future where capitalism would disappear. His musings on a possible work-less future from nearly a century ago can still inspire thought in the present.

These issues (on capitalism vs a post-capitalist future) cannot be fully addressed here. Instead, they require greater reflection and discussion, not least on how the economy might look without capitalism; however, they can be seen to reinforce the point made above that the question of AI (including its risks and threats for work) requires us to imagine different and radical futures. Beyond seeking more jobs under present capitalist conditions, we might instead see a way to reduce the importance of work in our lives, while also making the work we do more human. Now this would be a compelling and seductive vision for a more prosperous and virtuous future.

On this last point, a note of caution and realism can be added. While it is important to re-envision AI technology and the future of work and society, there are obvious barriers in realising any radical vision. The risk is that AI technology—given its present ownership and direction—will continue to thwart the goals outlined above. It may not eliminate undesirable work tasks and may replace the meaningful parts of jobs. It may also add to work hours. Barriers here include the power of capital owners to resist positive changes in work and to impose their own interests at the expense of those of workers.

There remain different attempts in the present to reimagine the future of work. Some of these are sceptical about the scope for AI to improve work and add to leisure time (Benanav 2020). These attempts, however, only underline the need to work out what a better future might entail and how it might be realised. While we can lament the present course of AI and cast doubt on its scope to improve well-being in the future, we can still aspire to redirect AI in ways that allow for a better quality of work and life.

## Political implications: post-work futures, UBI and work time reduction

A final set of considerations can be addressed. These relate to strands of research and political issues not already considered, at least head-on.

The first relates to ‘post-work’ futures (Srnicek and Williams 2015). These embrace automation directly as a way to abolish work. They side with a policy of ‘full automation’ and reject approaches such as that of Acemoglu and Johnson on the basis that they embed the prevailing work society. They arrive at this view by assuming that work is degrading and alienating. In the late David Graeber’s (2018) memorable words, most jobs are ‘bullshit’. The hope is then that AI can be used to release humanity from work. Reforms to achieve this outcome are seen to entail overturning the system of wage-labour.

The problem with the post-work literature is that it overplays the costs of work. It misses how work can have a positive place in people’s lives. This is not to succumb to some kind of ‘false consciousness’ idea where workers like jobs that are inherently alienating, but instead to recognise that work (under appropriate conditions) can help to enliven life. There is also limited recognition of the scope for technology to be used to realise the intrinsic benefits of work. Marx, for his part, thought that the technology developed under capitalism might be repurposed to reduce the alienation of work (Sayers 2005). His vision of a socialist future where people would relish work alongside enjoying free time stands in sharp contrast to the vision offered by modern post-work ideologues. Indeed, it offers a way to transcend it (Bellamy-Foster 2017; Spencer 2022).

The second consideration relates to the merits or otherwise of a UBI. Acemoglu and Johnson, again as was mentioned above, resist a UBI. This is partly because it cannot substitute for the benefits of work. As a result, they favour policies to preserve people in paid work. These policies are justified because of the positive contribution of work to people’s well-being.

As argued above, however, pro-work arguments like those of Acemoglu and Johnson miss the legitimate reasons that exist for reducing work and work time. A UBI might have a place in giving people the opportunity to gain a life with a reduced work commitment and hence may help to enhance human freedom and well-being. By itself, however, it would not remove the need for a focus on work reform. To the extent that a UBI would not eliminate work from society, then political and ethical questions will remain over its organisation and these questions can be seen to extend to the use of technology—in the latter case, to reduce alienating work and to raise the average quality of work. A UBI, in short, does not stop, but rather invite further discussion on ways to reform work, including via the use of AI.

A UBI, of course, faces other problems. It can be appropriated by the political right and used to block reforms, including in work (Jäger and Zamora 2023). It may also discourage movements out of the family and prevent meaningful participation in work and society (Honneth 2023). These and other problems only underline the need to see beyond a UBI in improving work and life conditions.

The third consideration is the case for work time reduction. There has been a lot of discussion recently about this case, particularly in relation to the case for a 4-day working week (Coote et al. 2020). This discussion has been accelerated by the COVID-19 pandemic, as attitudes to work have been revised. The prospect for AI to take more jobs has also prompted consideration of how work time might be reduced to accommodate the possible automation of work.

To return to the arguments made above, automation can be seen to be beneficial where it allows for more leisure time. The benefits for people may extend not just to more time with family and friends but also to shorter commute times and more sustainable forms of consumption that have positive outcomes for the environment (Schor 2005). Work time reduction may help the economy adapt to automaton while yielding wider benefits to individuals, society and the planet. Again, it is not a matter of pursuing it in isolation, but seeking to realise it alongside improvement in the quality of work. Based on these ideas, we might imagine a future where AI helps to set the foundations for an economy that is more thriving and sustainable, including from an ecological perspective.

## Conclusion

This paper has considered how AI might impact on the present and future of work. For some notable writers like Acemoglu and Johnson, there is a clear view on how AI should be used and how it should be directed. They call for AI to be used for the goal of complementing human labour. Believing that work is good in itself, they support measures to sustain people in paid work and reject a focus on automation. The reforms they recommend to achieve the above goal are far-reaching in nature—for example, they include opening up AI technology to greater democratic scrutiny and control.

Criticisms of the above approach have been made in this paper. These include the skewed focus on protecting paid work rather than on reducing it. The case for working less gets lost in a rhetoric that demands workers keep on working. Even though the focus is on work’s benefits, the scope for transforming work in more systemic ways is neglected. The vision of making work lighter—shorter in hours and higher in quality—is not taken at all seriously. This leads to a limited understanding of how AI might transform the way that both work and leisure are conducted and experienced in the future.

One implication of these criticisms is that the meaning of human-complementary technology needs to be expanded. It does not mean simply maintaining ‘good jobs’ but also seeking conditions where people can attain meaning in both work and leisure. Complementing human capabilities means respecting the needs of people for a life beyond work and automating work so that work hours can be reduced. It also means creating work that people can experience as meaningful. Achieving lighter work requires broader changes in ownership and control. It entails democratising workplaces. While Acemoglu and Johnson map out an extensive policy agenda, they do not commit to the kind of radical overhaul of work that is required to harness AI for a better and lighter future of work.

Another implication concerns the conception of a ‘human-centric AI’ that Acemoglu and Johnson and others support. At one level, this conception misses how different interests compete over the direction of AI—under capitalism, in particular, the interests of workers are forever hemmed in by the profit imperative, meaning that the use of AI can coincide with inferior outcomes such as long work hours and low pay. Capitalism, in essence, is antithetical to a ‘human-centric’ approach to AI. At another level, the above conception is too broad for genuine critical discussion. It makes the conditions for a ‘human-centric AI’ appear uncontentious (who would not find reason to support it?) when the reality is that AI is linked to a class-based politics. If the tensions in as well as possibilities for AI are to be more fully considered, then it would be perhaps better if the conception of a ‘human-centric AI’ was abandoned altogether.

This paper has also outlined implications for different ideas and policy measures. Post-work perspectives that focus on abolishing work neglect how the quality of work can be enhanced. Advocates of a UBI understate or overlook the need for work reform. Finally, from the perspective of enhancing well-being, it is important that due consideration is given to reducing work hours, including potentially via the harnessing of AI technology.

To conclude, AI presents both threats and opportunities for work. In truth, no one knows how exactly AI will develop—its effects on the quantity and quality of work remain to be determined. It is clear, however, that there is room to intervene and change the course of AI in ways that benefit the majority in society. Acemoglu and Johnson realise this fact, as do many other modern writers. Yet, as argued above, there is more to do (beyond the arguments made by these writers) in setting out the conditions and broader vision for a future where AI helps to improve the lives of people at work and beyond it. The point is that any radical redirection of AI towards the lightening of work will require fundamental change in the way that the economy is owned and governed.

## Data Availability

Not applicable.
